# Magnetoencephalography in Stroke Recovery and Rehabilitation

**DOI:** 10.3389/fneur.2016.00035

**Published:** 2016-03-31

**Authors:** Andrea Paggiaro, Niels Birbaumer, Marianna Cavinato, Cristina Turco, Emanuela Formaggio, Alessandra Del Felice, Stefano Masiero, Francesco Piccione

**Affiliations:** ^1^Laboratory of Neurophysiology and Magnetoencephalography, Department of Neurophysiology, Institute of Care and Research, S.Camillo Hospital Foundation, Venice, Italy; ^2^Institute of Medical Psychology and Behavioral Neurobiology, University of Tübingen, Tübingen, Germany; ^3^Section of Rehabilitation, Department of Neuroscience, University of Padova, Padova, Italy

**Keywords:** magnetoencephalography, stroke, connectivity, rehabilitation, brain–computer interface

## Abstract

Magnetoencephalography (MEG) is a non-invasive neurophysiological technique used to study the cerebral cortex. Currently, MEG is mainly used clinically to localize epileptic foci and eloquent brain areas in order to avoid damage during neurosurgery. MEG might, however, also be of help in monitoring stroke recovery and rehabilitation. This review focuses on experimental use of MEG in neurorehabilitation. MEG has been employed to detect early modifications in neuroplasticity and connectivity, but there is insufficient evidence as to whether these methods are sensitive enough to be used as a clinical diagnostic test. MEG has also been exploited to derive the relationship between brain activity and movement kinematics for a motor-based brain–computer interface. In the current body of experimental research, MEG appears to be a powerful tool in neurorehabilitation, but it is necessary to produce new data to confirm its clinical utility.

## Introduction

The introduction in the early 1980s of magnetoencephalography (MEG) recording devices boosted its clinical application: multichannel MEG provided a superior spatial resolution compared to electroencephalography (EEG) and the possibility of detecting dipoles tangential to the cortical surface were its main advantages. MEG was initially deployed in the presurgical evaluation of epileptic foci, given both the reliability in localizing superficial cortical epileptic foci (1) and the precise indications for placement of intracranial electrodes (2). It became subsequently obvious that processing of natural language is more accessible with MEG than with EEG or functional magnetic resonance imaging (fMRI) because the magnetic field changes can be more precisely free from noise and artifacts (3). The high variability in the localization of frontal and parietal language processing sources creates considerable difficulties for the neurosurgeon to discriminate between eloquent areas involved in speech and language and “silent” brain tissue, so that the removal of tumors and other malformations of the brain and its vasculatum becomes a challenging operation. The combination of MEG and structural MRI provides the optimal solution to this problem because of the small fiducials positioning and localization errors (i.e., approximately 2 mm) assuring a reliable coregistration of functional and structural data (4).

With the installation of the new generation MEG having more than 250 sensors able to provide even further improved spatial resolution and accessibility of source localization algorithms (see below) to deeper brain structures and cerebellum, MEG technology has been successfully introduced to resolve the more complex problems of ­recovery and brain reorganization after stroke and other types of brain injury. Particularly, recovery prediction and assessment has become the focus of interest in clinical use of MEG in rehabilitation.

Magnetoencephalography has maintained part of its advantages even after the introduction of high-density EEG, consisting of a spatial sampling up to more than 250 electrodes. Although signals detected by the two recording techniques appear to be generated by different limbs of the same circuit, recent studies (5–8) have suggested that they have at least partially distinct generators. Indeed, MEG is particularly sensitive to activity originating in the cortex directly underlying sensors and is insensitive to radial dipoles, whereas EEG seems to reflect volume conducted activity and is sensitive to radial and tangential dipoles (9). Thus, the two techniques should be considered mutually complementary rather than mutually exclusive.

Finally, the rapid development of non-invasive Brain–Machine Interface Research [BMI or also termed brain–computer interfaces (BCI)] during the last 10–15 years (10–12) has launched a completely new and challenging field of application to MEG technologies: on-line recordings from selected MEG–sensor combination has been used to drive exoskeletons and computer switches for therapeutic purposes (see below). With BMI research, MEG has been transformed from a passive recording and documentation/diagnostic device into an active treatment and rehabilitation instrument (13).

The success of BMIs has reactivated the tradition of neurofeedback research, popular in the EEG community from the 60s–80s of the last century (14). MEG allows simultaneous observation and self-control of extremely specific localized dynamic sources of neuromagnetic activity together with widespread, more general, brain activity changes. In addition, the availability of fast computing algorithms for providing feedback of dynamic connectivity changes has introduced a new area of interest for directly manipulating changes and the related functional connectivities of oscillatory brain activity. When such algorithms allow modeling of oscillatory sources’ directionality, the effective connectivity can be estimated by describing how anatomically connected areas interact with each other (15).

## MEG Source Localization in Neurophysiology

The correct identification of the sources responsible for producing the observed brain activity is a fundamental goal in neurophysiology, both for diagnosis and treatment planning. In recent years, the accuracy of spatial localization in MEG has improved considerably (16). Despite that, difficulties in location are related to physical limits in the spatial resolution of MEG, the so called “inverse-problem” (17–23).

Distributed source models consider that the dipoles are regularly distributed in cerebral volume according to a 3D grid and each solution point is considered as a possible location of a current source, thus there is no *a priori* assumption on the number of dipoles. Unfortunately, an infinite number of distributions of sources within the 3D grid can lead to exactly the same scalp potential map (the ill-posed inverse problem).

Among many others, one way to partially solve the electromagnetic inverse problem is the widespread technique based on minimum-norm estimation (MNE). Solutions based on MNE assume that the 3D current distribution should have minimum overall intensity (smallest L2 norm), and it requires minimal hypotheses and has nevertheless a reasonably good localization accuracy in representing current sources as active areas (24, 25).

Another method based on current density distribution is, for example, the Low-Resolution Brain Electromagnetic Tomography (LORETA) (26). This algorithm introduces additional constraints selecting the solution with a smooth spatial distribution by minimizing the Laplacian of the weighted sources. Also in the case of current density methods, for a sufficient signal-to-noise ratio, operations are typically conducted on averaged data sets (evoked activity).

In Beamforming methods, the goal is not trying to explain the whole measured fields, but rather to estimate the contribution of a single brain position of interest of the observed field (27). Furthermore, a beamformer is based on the spatial covariance of the source electrical activity, rather than on its strength, and it is applied on raw data sets because it does not need of averaged datasets of evoked responses. It can be used to analyze induced brain processes, and it does not require neither an *a priori* specification of the number of active sources nor information about their geometry. As a drawback, beamforming methods are blind for time correlated neural activity (19, 21, 22, 28).

A beamformer is a versatile type of spatial filter set to transmit or receive signals preferentially in some directions over others. A beamformer amplifies signals from different locations by different weights, according to the desired sensitivity pattern, to promote the contribution of signals coming from a specific direction, while attenuating signals from other locations. A main lobe in the direction of the signal of interest preserves the informative content, whereas nulls and sidelobes suppress noise and interference signals. In fact, the word “beamforming” actually refers to the typical profile of early spatial filters, whose polar plot design reminds the shape of a “pencil beam” (18, 28).

The worth of beamforming technique, and of spatial filtering in general, is the ability to discern and separate signals originating from different locations even though they may present an overlapping frequency content. Where temporal filtering cannot be used to distinguish an interference signal having the same temporal frequency band of the desired signal, spatial separation can be exploited to effectively reject the undesired content (22). Beamforming is the spatial analogous to frequency domain analysis of time signals: while in time-frequency filtering, the frequency content of a time signal is represented by its Fourier transform, in spatial filtering, the angular (directional) spectrum of a signal is reconstructed via a Fourier analysis of the way signal reaches different parts of the set of sensors.

Many researchers have tried to deal with the inverse problem using beamforming techniques. For instance, van Drongelen et al. (18) demonstrated that the resolving power of the technique increases with the number of available sensors and with the SNR, while it gets worse as the source gets deeper. Quraan and Cheyne (24) found that degradation in beamformer performance is the presence of sources with high temporal correlation.

In view of the many different existing source localization methods, the most crucial question remains the choice of the optimum method, in order to have the most correct solution. The main problem is the difficulty of obtaining evidence about the true location of the sources, since there is no clear established gold standard that allows judging the goodness of the result of the different inverse solutions (29). For this reason, many studies evaluated and compared source localization algorithms through simulations, concluding that the optimum method does not exist but each source modeling algorithm has its own strengths and limitations.

**Figure 1:**
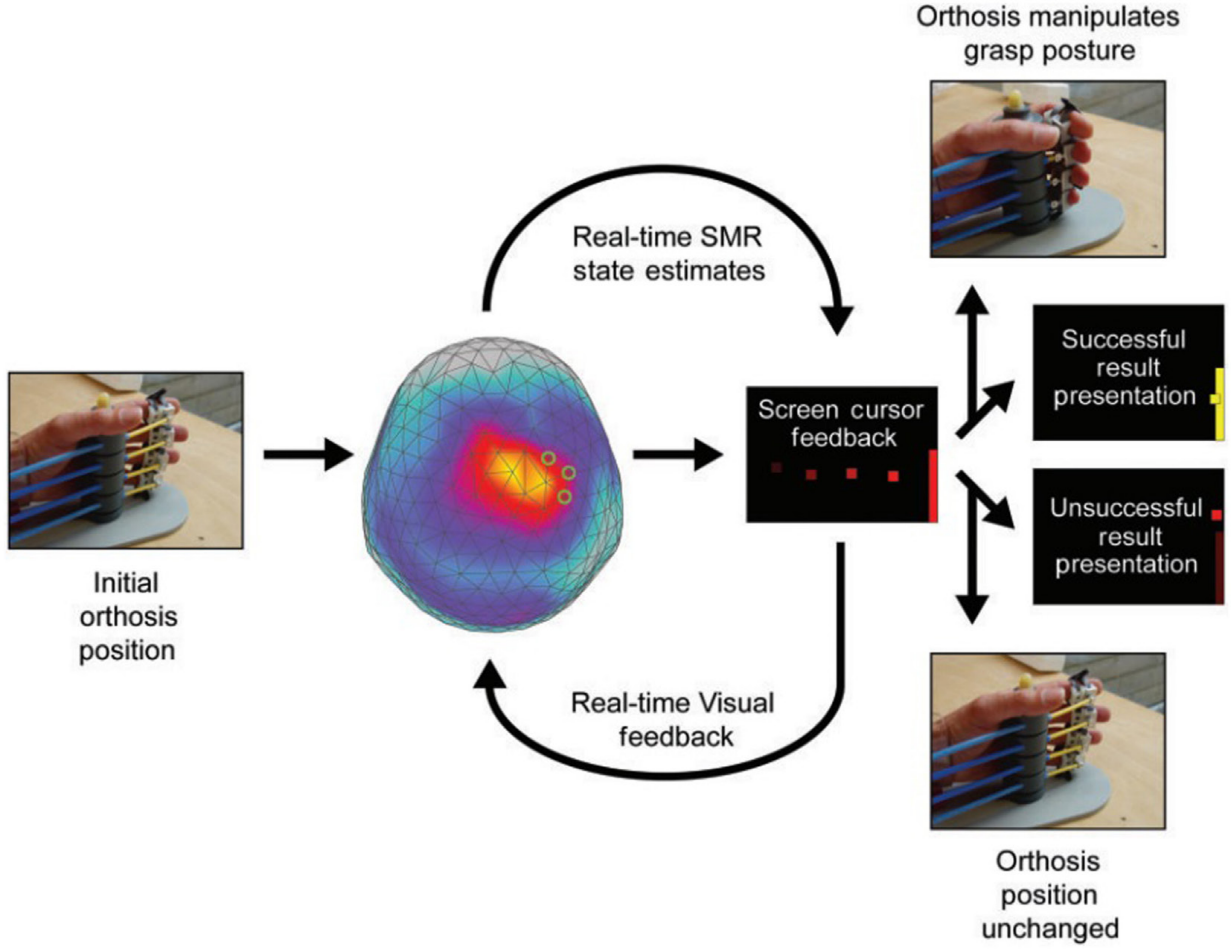
**Example of a trial for sensorimotor rhythm (SMR) modulation through grasping imagery training**. Whole-head magnetoencephalography data were continuously recorded throughout each training block [Buch et al. (32)].

Algorithms mentioned above localize time-locked brain activity, except for LORETA used to compute source from EEG cross-spectra. More advanced MEG source reconstruction methods, as beamformer, have been recently developed, capable of localizing both time-locked and induced oscillatory activity from brain regions. The beamformer, implemented by synthetic aperture magnetometry algorithm, has been shown to be able to successfully detect event-related desynchronization/synchronization (ERD/ERS) in the beta and mu frequency bands in the sensori-motor areas during median nerve stimulation (30) and voluntary hand movements (31). Given the importance of the sensorimotor system in stroke recovery, these studies highlight the relevance of this source localization method to identify precise frequency changes in sensorimotor cortical oscillations (Figure 1).

## MEG Studies on Brain Reorganization and Rehabilitation after Stroke

Stroke is the leading cause of long-term disability among adults, and there is no consensus on how to treat residual disability (33, 34). At an European regional level, the estimated stroke prevalence of at least 1 million stroke events per year and the estimated incidence of 1.5 million cases per year in 2025 (35, 36) make the relevance of an effective rehabilitation crucial for both social and economic burden. Major efforts have been devoted in order to find the better ways to improve long-term stroke outcome, especially in the motor and cognitive realm. Nonetheless, a number of patients experience insufficient or partial recovery; 30–66% of chronic stroke victims are resigned to live with significant functional and/or cognitive deficits (37–40). The development of new effective rehabilitation strategies relies on a better understanding of the mechanisms underlying functional recovery. In this, perspective novel advancements in neuroimaging and neurophysiology techniques are of paramount importance to make approaches to stroke rehabilitation better and more effective (33, 34, 37). Single or combined non-invasive techniques to study brain function, including fMRI, positron emission tomography (PET), transcranial magnetic stimulation, transcranial direct current stimulation, near-infrared spectroscopy, EEG, and MEG contributed to the debated issue of the different recovery potentials of poststroke individuals. Although there is not a technique significantly better than others, each one has particular feature. Combined recordings as in EEG–fMRI have the dual advantage offered by the high temporal resolution of EEG and the spatial resolution obtained with neuroradiological exams. In the EEG–fMRI coregistrations, EEG is analyzed to obtain regressors of interest used in the common General Linear Model framework. However, fMRI is intrinsically limited by the hemodynamic response which extends across several seconds. MEG directly measures cortical neural activity, and unlike blood oxygenation level-dependent (BOLD) signal measured by fMRI, it is unaffected by neurovascular uncoupling. Moreover, modified vasomotor reactivity in stroke easily affects the BOLD hemodynamic response but leaves the MEG signal intact.

Magnetoencephalography signals can be efficiently interpreted by different analytical approaches that allow to quantify contributions of specific brain areas and consequently to explore the functional and structural spontaneous reorganization of brain networks in stroke patients (11, 41–44). MEG with its high density of sensors and its real-time resolution is one of the main technology allowing the mathematical reconstruction of these network properties.

Recently, a complex network analysis, known as graph theory, has become progressively more and more widespread in brain research as an appropriate model to describe both structural and functional connectivity (45). Direct and indirect, as well as intrinsic and extrinsic, interactions between different brain regions are in fact explicitly depicted by means of nodes and edges depending on their strength (46).

A network, mathematically represented by a graph, can be characterized by several measures. Many studies suggest that one of these properties, cost-efficiency, is an important optimization principle that governs both structural and functional brain network architecture (44). Highly cost-efficient networks preferentially employ long-range connectivity, allowing faster and more robust information transfer between discrete brain regions, minimizing the related energy cost of fiber pathway maintenance (47, 48). Achard et al. (49) and Bassett et al. (50) showed that this peculiarity also correlates with behavioral aspects: in both healthy volunteers and patient groups, in fact, memory and intellectual performance result to be overseen by a cost-efficient concept.

Another fundamental characteristic of network graphs emphasize brain regions or white matter fiber pathways that play a crucial role to promote functional integration between remote brain areas (51, 52). As a proof, Wang and colleagues found that this property predicts motor hand recovery in stroke (11). Furthermore, betweenness centrality has been proven to highlight how functional dynamics of a certain network region are susceptible to the effect of brain lesions and injuries (53, 54).

Cost-efficiency and betweenness centrality are, thus, two features of brain networks allowing to investigate any possible correlation between anomalous network activation patterns and the outcome of behavioral rehabilitation following stroke (55). Another great benefit of these two properties, especially when compared with voxel-based techniques, is their description within a topological framework and the chance to find non-spatial relationships between them, which is useful for dealing with an heterogeneous stroke population and, more in particular, for describing the pathological changes in brain connectivity patterns relatively to different brain damages (42, 51).

Another valuable descriptor of brain interactions, the effective connectivity, can be inherited from neuroimaging techniques to MEG recordings after proper adaptation. It constitutes a different method for brain networks identification as it mainly relies on directionality of the information flow between brain regions (i.e., how much a cortical area exerts control over a different cortical area).

Effective connectivity can be mapped by frameworks using phase synchronization algorithms or algorithms in the context of Granger Causality (that quantifies the usefulness of unique information in one of the time series in predicting values of the other, such as directionality phase index, direct transfer function or partial directed coherence) based on oscillatory properties of analyzed source data (15, 56, 57). These new techniques allow to replicate – by MEG – some relevant fMRI findings concerning poststroke motor network effective connectivity and its relationships with functional recovery (42, 45, 58, 59). Indeed, recent studies reported that the degree of network disorder and functional deficit after stroke is mainly caused by a significantly reduced intrinsic neural coupling between higher order premotor and motor areas and suggested that the Granger causality measures of network information flow can be used as a reliable biomarker for evaluating rehabilitation in stroke survivors (60).

As regards the classic stroke rehabilitation, interventions are largely unsuccessful in recovering the most severe poststroke motor impairments (61). This lack of improvement is particularly evident for hand function (62): reaching and grasping are defined and performed by activating specific neuronal populations interconnected into functional networks (63). If some of the anatomical components of these networks are compromised (e.g., by stroke), functional dynamics impair (53, 54). After the acute event, the peri-infarct regions come into a state of synaptic instability lasting a limited time window in which compromised networks are rapidly reorganized (12, 64–67). For instance, an enlargement of the somatosensory affected area after stroke has been reported, although a renormalization of upper limb area seems to be related to the degree of functional recovery at follow-up (68). When sub-acute phase is reached, these networks can reach a stage where capacity for reorganization gets weaker. Indeed, even during the chronic phase a considerable plasticity is maintained (66, 69–71). The elapsed time from stroke onset seems to play a crucial – and still partially unknown – role on residual resilience of subjects (38, 41). Studies investigating poststroke motor recovery (in sub-acute and early chronic stages) by means of fMRI found different intrahemispheric and interhemispheric effective connectivity alterations within the motor network mainly involving primary motor areas, premotor cortex, and supplementary motor areas (57).

There is no consensus on the precise mechanisms underlying functional recovery after stroke. Three main processes have been identified: changes at molecular and cellular levels taking place in the peri-infarct and remote brain areas, involvement of contralateral homologs via the unmasking process activating previously inhibited connections, and the recruitment of other compensatory brain areas (72).

The majority of studies states that functional recovery after stroke mostly occurs through reorganization of cortical activity in the proximity of the infarct or its connected areas. Recent fMRI-based research studies shed light on the reorganization role of effective connectivity among the motor network’s components (both intrahemispheric and interhemispheric) emphasizing the relevance of nearly distal cortical areas in the poststroke brain reorganization process (41, 58, 59). Sleep, through neuroplastic and encoding processing mediated mainly by slow waves sleep (SWS) and sleep spindles, plays an active role in this recovery process. Indeed, the finding that sleep spindles of stroke patients have a reduced amplitude as well as a reduced cortical activation than healthy subjects (73, 74) and that SWS remains lower over the perilesional area (75) supports the fundamental role of sleep in recovery.

Nonetheless, evidence suggests that recovery of motor function may involve modifications of intracortical wiring patterns as well. One plausible consequence of these novel wiring patterns is the recruitment of compensatory areas of the brain that may not be directly related to the damaged area.

This brief overview confirms that, despite multiple mechanisms may exist, the recovery process after stroke mainly depends on the degree of involvement of unaffected areas, whether they are proximal, distal, or even contralateral to the lesion. Thus, the investigation of poststroke brain plasticity with the help of the available brain-mapping techniques is fundamental to reveal the recovery dynamics due to natural processes or as a consequence to therapy and specific training programs (37).

Recent studies agree that learned non-use of the paralyzed limbs coupled with overuse of the healthy (contralesional) limb expands cortical representation and excitability of the contralesional healthy projection area, as well as that of their associative secondary and tertiary regions (76). The ensuing neurophysiological phenomenon is an imbalance of the primary motor cortex (M1) excitability. This phenomenon causes a relative hypo-excitability in the stroke-affected hemisphere and relative hyper-excitability in the contralesional hemisphere, with worse clinical outcomes for patients with greater imbalance. Rebalancing of cortical excitability in patients with stroke has been associated with improved upper limb function (57, 77–81). Constraint movement therapy (CMT) (82) seems to reactivate the paralyzed body parts in chronic stroke patients through immobilization of the healthy limbs and increased use and somatosensory feedback from movements of the plegic limb. This leads to substantial reorganization of the peri-lesional areas as documented before and after CMT with MEG and fMRI (82–84). However, CMT is not applicable in patients (one-third of all chronic stroke patients) with no residual limb movement because the complete paralysis does not allow to use the remaining non-constraint side of the body for functional activities. In these severely affected individuals, direct modification of the described imbalance between the two hemispheres through training and/or brain stimulation is mandatory, and opens up bright perspectives for rehabilitation and its assessment with MEG that can detect changes in spontaneous oscillatory activity measured by spectral analyses, one of the most prominent indices of tissue dysfunction.

Magnetoencephalography has also been used to study some of the neuropsychological deficits following a stroke. Unilateral spatial neglect (USN) is a characteristic failure to explore the contralateral space of a brain lesion and is probably related to high-order attentional deficits affecting lower-order (early) sensory processing (85, 86). MEG has been deployed to measure visual evoked magnetic fields (VEFs) that are disrupted in patients with USN, supporting the concept that deficits in visual processing differ according to the clinical subtype of USN and the lesion location (87).

Magnetoencephalography might also be of help in the evaluation of other rehabilitative approaches (88, 89). Action observation modulates activation of the viewer’s motor (90) and somatosensory (91) cortex, with stronger motor cortex effects for live than video presentation (92). Measures of functional activity acquired with MEG while hemiplegic patients are imagining, observing, and executing simple movements have higher accuracy than fMRI in the selection of good responders to motor rehabilitation. By applying frequency-domain beamforming to whole-head MEG data, neuronal plasticity associated with a motor training program combined with sham stimulation and peripheral neural stimulation (PNS) was studied in a case series of chronic stroke participants (93). The key findings were a reduction in beta synchronization during and after-movement and a reduction of gamma synchronization in the affected primary motor cortex and supplementary motor areas following motor training. In addition, the posttherapy decrease in gamma synchrony was significantly stronger in the affected precentral gyrus of subjects receiving PNS than to the sham group. These reductions in cortical synchronization may indicate that the intervention brings inhibitory function back toward more homeostatic levels, possibly by modulating stellate cell firing rates or local synaptic connectivity, thereby enhancing network efficiency in motor cortices.

Magnetoencephalography data analysis and source reconstruction were also applied to investigate the effects of motor imagery (MI) on rehabilitation. MI substantially activates the same cortical areas firing during active movement (94, 95), but whether the activated cortical areas are the same is still unknown. A recent study addresses this topic by defining neural correlates of real and imagined finger movement (96, 97). In considerable accordance, both MEG and fMRI data showed a significant overlap between brain activation during MI and real movement. On the other hand, Burianová and colleagues noticed that whereas sizeable differences between MI and motor execution in the brain areas involved in visuospatial processing (e.g., left inferior parietal lobule, parahippocampus, right superior temporal gyrus and superior frontal gyrus), clearly activated in movement imagination, MI seems not able to yield the activation of areas related to somatosensory coordination (i.e., right primary motor and sensory cortices, cerebellum, putamen, and posterior parietal areas) as active movement does.

Other authors focused on the application of different neurorehabilitation techniques in stroke subjects, such as the mirror therapy (97). Mirror therapy consists of the attempt of synchronously perform bilateral hand movements while observing the mirror reflection of the unaffected limb in the position of the affected limb. Ten subjects with poststroke upper limb impairment and 13 healthy controls underwent this protocol during MEG recording. Interestingly, beta-band ERD was symmetrical during bilateral movement and unaltered by the mirror condition in controls. In stroke subjects, ERD was reduced compared to controls, but an asymmetry emerged with a greater response over contralesional compared to ipsilesional motor cortex. This initial asymmetry in movement-related beta desynchronization between hemispheres was made more symmetrical by the presence of the mirror. These data seem to provide the neurophysiological rational for mirror therapy effectiveness in stroke individuals.

Other neurorehabilitation techniques incorporate musical cues to facilitate motor recovery in poststroke individuals [e.g., rhythmic auditory stimulation (RAS) or music-supported therapy (MST)]. In general, musical training leads to reorganization of brain function and structure related to sensorimotor, auditory, and visual information processing. A recent paper demonstrated in three poststroke subjects that paced tones (metronome) induced a similar beta-ERD over sensory–motor areas both during listening and during finger tapping at the same frequency, as recorded by MEG (98). In addition, desynchronization dynamics in both conditions was further modified after 5 weeks of MST training, stressing the neurophysiological similarity between paced motor activity and rhythmic musical stimulation on brain plasticity.

Likewise in motor outcomes, MEG potentiality is exploited also in subjects with aphasia following speech therapy in order to give better predictive information about the cognitive neuropsychological approach or the intensive language treatment and to optimize the rehabilitation process. MEG has been demonstrated to be sensitive to neurophysiological changes related to rehabilitative therapy for stroke-induced aphasia (99–101). Functional imaging provides discordant results on the correlation between brain activity patterns, language function, and recovery after aphasia rehabilitation therapy. On one hand, increased activation in the right hemisphere after therapy appears to be related to language function recovery (102, 103), whereas an increased activity in the dominant hemisphere (104–106) or even bilateral increases in activation (107–109) have been reported as related to behavioral improvements. In addition, increased activity in peri-infarct regions of the dominant hemisphere was singled out as an indicator of positive linguistic outcome (100).

## MEG–Brain–Machine Interfaces in Rehabilitation of Stroke

A recent consensus paper [Cumberland Consensus (110)] tried to describe the reasons for the lack of transformation of basic neuroscience research in stroke into novel clinical practice. So far, animal and *in vitro* experiments (with the exception of Taub’s Silver Spring monkey research which led to CMT) have not improved rehabilitation practice, and the distance between basic neuroscience academic research and clinical practice attending to the patients’ needs has become large and rarely controlled clinical studies can compensate.

**Figure 2:**
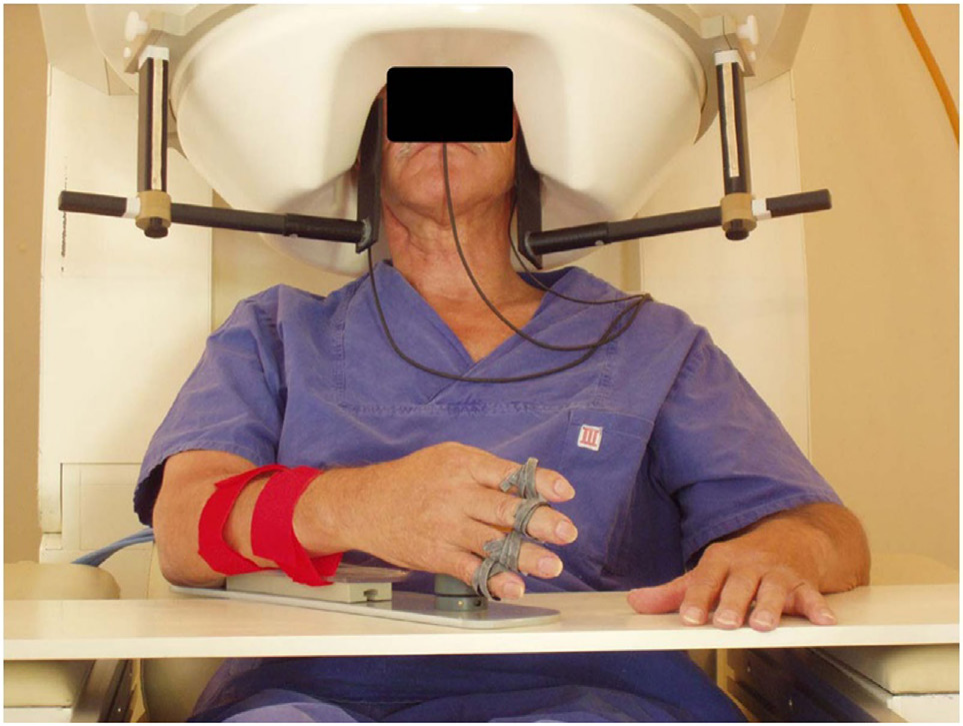
**MEG-BCI: hand orthosis controlled by ipsilesional central mu-rhythm [modified from Birbaumer and Cohen (114)]**.

However, this picture is changing thanks to the introduction of BMI-training programs for severely affected chronic stroke patients studied with MEG (111–114) (Figure 2). For instance, a 1-year longitudinal follow-up of a stroke case reported a mixed MEG and EEG BMI-based training combined with physical therapy (112, 113). After 1 year, the upper limb function as well as gait parameters improved significantly, coupled with an increase of micro-oscillations in the ipsilesional motor cortex.

One-third of chronic stroke survivors do not improve even after intensive physiotherapy or robot-based rehabilitation. This group constitutes the target clinical population for BMI rehabilitation. Dimyan and Cohen (12) used neuromagnetic-BMI in eight subjects with complete hand paresis without any residual movement after subcortical strokes. Patients were trained to desynchronize the ipsilesional sensorimotor rhythm (SMR) estimated by MEG data and successful desynchronization moved the paralyzed hand affixed to an orthosis in the MEG (12). Six out of eight patients learned within 20 sessions to move the orthosis based on SMR-magnetic fields in more than 80% of the trials. However, the successful control of the BMI in the MEG-environment did not generalize outside the laboratory: grasping functions remained unchanged.

**Figure 3:**
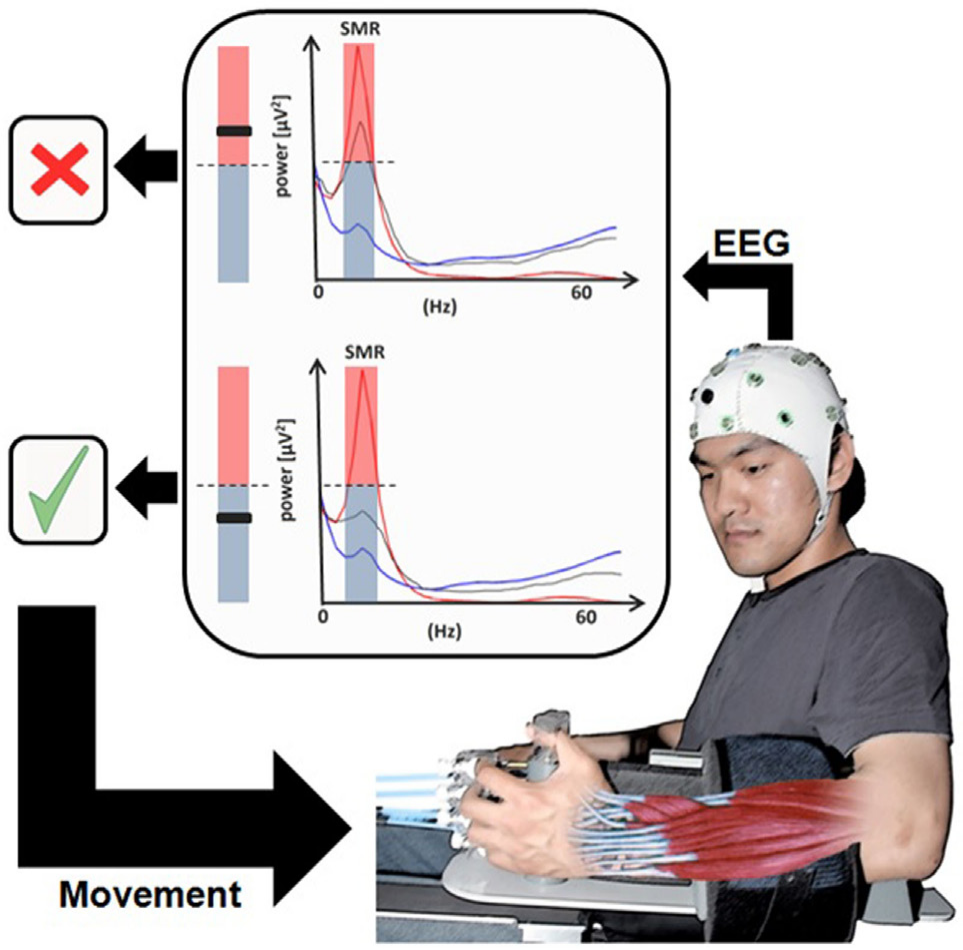
**Brain–machine Interface in paralyzed chronic stroke patients’ rehabilitation**. User wearing an EEG system with the hand attached to the orthosis to drive extending fingers. The sensorimotor rhythm power recorded from the ipsilesional electrodes (gray line) is translated into movement of the orthosis [modified from Ramos-Murguialday et al. (84)].

In a reanalysis of these MEG data, the authors demonstrated that residual functional network integrity at the ipsilesional side and intact connectivity between the posterior parietal visuomotor fiber tracts, particularly the ipsilesional superior longitudinal fascicle and the fronto-motor areas, determines the learning of the BMI-control skill (56). These discovery led to the first clinically successful controlled BMI study in chronic stroke with no residual hand function (84) (Figure 3). This study used EEG-derived signal, following exactly the MEG protocol of Buch et al. (13). In fact, after each BMI-session subjects had to train the same movement sequence used during the BMI session without the BMI assistance in a natural, non-laboratory environment, thus reinforcing motor behavioral skills and patients’ motivation. The control group received exactly the same treatment except that during BMI sessions, the passive movements induced by the BMI were not contingent on the patient’s brain activity, and SMR occurred randomly. After 20 sessions of BMI combined with behavioral physiotherapy, only the experimental group (*N* = 16) with contingent BMI training showed substantial changes in the degrees of freedom of skilled movements and cortical reorganization measured with fMRI: after training, the center of brain activity shifted from the healthy, contralesional hemisphere to the central ipsilesional hemisphere.

This impressive result clearly indicates that the decisive mechanism of neuroplastic changes and behavior depends on the learned contingency between movement intention (measured by SMR-desynchronization) and immediate (proprioceptive and visual) feedback from the BMI-induced movement. However, it also demonstrates that generalization of the learned effect outside the lab depends upon explicit generalization training. Therefore the EEG/MEG-based contingent BMI is crucial and necessary for the improvement in chronic stroke survivors without residual movement capacity (11, 109, 115).

The findings of Ramos-Murguialday et al. (84) even suggest the implementation – in a BMI context – of a very close intention-feedback contingency (within tens of milliseconds) instead of a more delayed contingency (one or more seconds) in order to reestablish the sensorimotor feedback loop disrupted by stroke. Such beneficial contingency seems to depend on a time-constrained basic neural mechanism underlying long-term synaptic potentiation (i.e., achievable when afferent and efferent synaptic stimulation coincides to induce spike-timing-dependent plasticity). It is very similar to the peripheral/central Paired Associative Stimulation (PAS) (115) acting at cellular level and already demonstrated *in vitro* (116) and *in vivo* (117). This close-to-real-time learning-potentiation training can be implemented by – but not limited to – the combination of operant conditioning based strategies and MEG technology, again because of its high-time and high-spatial resolution [for a review of learning strategies used in BMI field see Silvoni et al. (118)]. A proof-of-principle case report exploiting this neural mechanism, describing primarily the methodology of an EEG-based BMI-system without relevant clinical results, proved the technical feasibility of the very close intention-feedback contingency implementation (119).

An MEG-based BMI training can also be designed with different neural signatures of movement intention (i.e., SMR ERD) such as functional or effective connectivity between cortical areas belonging to the motor network. For instance, a pilot study demonstrated that real-time volitional control of neuromagnetic coherence can be gained in few hours (119). This study encourages new real-time brain connectivity investigations, in particular those related to movement planning, preparation, and execution, thus helping a better understanding of the motor network behavior and its dynamics in different stages of poststroke rehabilitation.

Finally, different mathematical models feeding the system can provide different results. A BCI modeled on MEG signal provided evidence in four-stroke subjects that the combination of a homogeneous reference value for ERD detection with graded feedback based on ERD strength leads to better BMI performance and learning than heterogeneous reference values with binary feedback. Thus, this training strategy may offer a better way to improve modulation of ipsilesional activity in the context of restorative BMI use in neurorehabilitation (120).

## Conclusion and Future Prospects

Magnetoencephalography appears to be a powerful tool both for the neurophysiological assessment of poststroke individuals and for the implementation of BCI rehabilitation methods. The advantage of a reliable source reconstruction via devoted algorithms provides an additional advantage of these techniques.

As a drawback, MEG is unfortunately not yet of common use, possibly due to the elevated costs and the need of a dedicated multidisciplinary team. Nonetheless, MEG presents advantages over EEG signal – i.e., the reliable reconstruction of also deep sources – pointing to a combined use of MEG and last generation high-density EEG.

The effort of the scientific community should thus be addressed to design good quality, large trials to properly develop the use of MEG not only as a diagnostic tool but also as a support in BCI.

## Author Contributions

Mr. AP gave important support in conception and writing of manuscript. Prof. NB gave his expertise in matter of MEG, stroke rehabilitation, and brain–computer interface. Dr. MC gave her support in the revision and of manuscript. CT gave her support in term of MEG technical parameters. EF followed the part related to algorythm and mathematical tools for MEG. Dr. SM and AF gave their support in the topic of neurorehabilitation. Dr. FP gave his knowledge in the field of stroke rehabilitation.

## Conflict of Interest Statement

The authors declare that the research was conducted in the absence of any commercial or financial relationships that could be construed as a potential conflict of interest.
